# Mathematical modelling of the growth of human fetus anatomical structures

**DOI:** 10.1007/s12565-016-0353-y

**Published:** 2016-07-08

**Authors:** Krzysztof Dudek, Wojciech Kędzia, Emilia Kędzia, Alicja Kędzia, Wojciech Derkowski

**Affiliations:** 1 Faculty of Mechanical Engineering, Wroclaw Univeristy of Science and Technology, Wroclaw, Poland; 2 Non-public Higher Medical School in Wroclaw, Wroclaw, Poland; 3 Department and Clinic of Anesthesiology and Intensive Therapy, Wroclaw Medical University, Wroclaw, Poland; 4 Public Higher Medical Professional School, Opole, Poland

**Keywords:** Human foetus, Growth curve, Gestational age, Mathematical modelling

## Abstract

The goal of this study was to present a procedure that would enable mathematical analysis of the increase of linear sizes of human anatomical structures, estimate mathematical model parameters and evaluate their adequacy. Section material consisted of 67 foetuses—rectus abdominis muscle and 75 foetuses- biceps femoris muscle. The following methods were incorporated to the study: preparation and anthropologic methods, image digital acquisition, Image J computer system measurements and statistical analysis method. We used an anthropologic method based on age determination with the use of crown-rump length—CRL (V–TUB) by Scammon and Calkins. The choice of mathematical function should be based on a real course of the curve presenting growth of anatomical structure linear size *Ύ* in subsequent weeks *t* of pregnancy. Size changes can be described with a segmental-linear model or one-function model with accuracy adequate enough for clinical purposes. The interdependence of size–age is described with many functions. However, the following functions are most often considered: linear, polynomial, spline, logarithmic, power, exponential, power-exponential, log-logistic I and II, Gompertz’s I and II and von Bertalanffy’s function. With the use of the procedures described above, mathematical models parameters were assessed for V-PL (the total length of body) and CRL body length increases, rectus abdominis total length *h*, its segments *h*I, *h*II, *h*III, *h*IV, as well as biceps femoris length and width of long head (LHL and LHW) and of short head (SHL and SHW). The best adjustments to measurement results were observed in the exponential and Gompertz’s models.

## Introduction

Medical literature analysis reveals that foetal growth assessment requires construction of mathematical models that may be extrapolated out of the observation period. This problem is poorly discussed in available literature (Sztencel and Żelawski 1984). This may result from scarce foetal material as well as the rare combination of morphological sciences and mathematics. Foetal period is still poorly recognized. Our own studies (Dudek et al. 2014; Kedzia et al. 2010a; 2011a, b, 2013a, b; Woźniak et al. 2012, 2014) have enabled the assessment of foetal structures by geometric dimension increase curve. Neither sexual dimorphism nor asymmetry was very characteristic. Other observations based on less material comprising a smaller age span (Badura et al. 2011a, b; Grzonkowska et al. 2014; Szpinda et al. 2011, 2013) revealed similar results.

The goal of this study was to present a procedure allowing human anatomical structure linear measurements analysis that arrived at mathematical model parameter estimation and evaluation of its adequacy. Theoretical discussion was substantiated with examples including body length, rectus abdominis muscle length, as well as length and width of femoral biceps of foetuses belonging to the Normal Anatomy Dept. of the Medical University of Wrocław (Kędzia et al. 2010, 2012). Our own examinations presented mathematical model structure algorithms of foetal structure growth (Dudek et al. 2014).

### Mathematical modelling

A physical object model is constructed on the basis of physical quantities describing the object's qualities. There are dimensions of three types:

- Input sizes *x*_1_, *x*_2,_ *···*,*x*_*j*_ (stimulations) regarded as the causes (e.g., age of foetus − *t*);
- Output sizes *y*_1_, *y*_2,_ *···*, *y*_*k*_ (responses) regarded as results (e.g., foetal structures geometric sizes–*y*);
- Influent sizes *w*_1_, *w*_2_ *···*, *w*_*l*_ describing environmental influence on the modelled object (e.g., foetus sex, mother’s height and weight, race).

Input and output sizes are strictly connected with the model formula:

$${\mathbf{F}}\left( {{\mathbf{x}},{\mathbf{y}},{\mathbf{b}}} \right) \, = {\mathbf{0}}$$

where: **x** = [*x*_1_ *x*_2_ … *x*_j_]^T^—stimulus vector (in the analysed case-single-element vector *x*_1_ = *t* (foetus life time -weeks);

**y** = [*y*_1_ *y*_2_ … *y*_k_]^T^—response vector (anatomical structures geometrical sizes *Y*_*k*_);

**b** = [*b*_1_ *b*_2_ … *b*_*m*_]^T^—vector of model parameters;

**F** = [*F*_1_ *F*_2_ … *F*_k_]^T^—vector of operators;

**0** = [0 0 … 0]^T^—vector formed of *k* zeros;

T—symbol of matrix transposition.

The selection of mathematical function should present the real course of *Y* size growth curve in subsequent weeks *t* of pregnancy. Many functions can be used for size–age interdependence; however, the following are most often considered (Jaworski et al. 1992; Kędzia et al. 2010; Muciek 2012).

1. Linear (Fig. 1a): (this model assumes a size stable growth rate for the whole period of pregnancy):
  $$Y = b_{0} + b_{1} \cdot t.$$

**Fig. 1:**
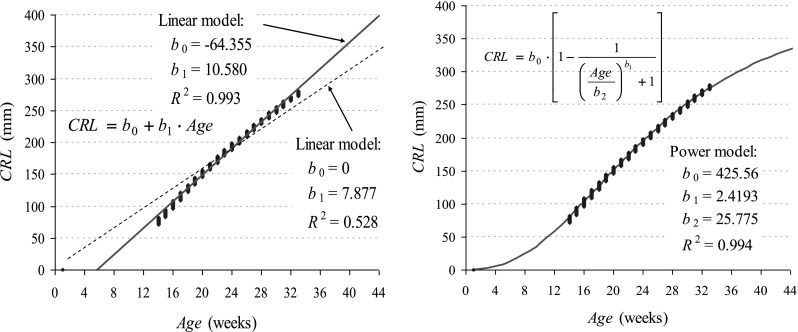
Monoequation, biparametric models of crown-rump length (CRL) growth vs. foetal sizes in Scammon’s and Calkins’ tables
2. Segmental-linear (Fig. 3): in this model, size–time dependence is presented with the use of at least two segments of various inclinations. For a three-equation model, independent variable limit values *t*_I_ and *t*_II_ should be established, and the model parameters should be estimated:
  $$\begin{array}{*{20}l} {y_{\text{I}} (t) = b_{{0{\text{I}}}} + b_{{1{\text{I}}}} \cdot t,\quad \;\quad t \le t_{\text{I}} } \hfill \\ {y_{\text{II}} (t) = b_{{0{\text{II}}}} + b_{{1{\text{II}}}} \cdot t,\quad \;t_{\text{I}} \le t < t_{\text{II}} } \hfill \\ {y_{\text{III}} (t) = b_{{0{\text{III}}}} + b_{{1{\text{III}}}} \cdot t,\quad \;t \ge t_{\text{II}} } \hfill \\ \end{array}$$
  The authors use the following procedure for three-segment linear function parameter evaluation:
  Estimate linear model parameters for the whole range of measurement data (*b*_0_ and *b*_1_).
  Estimate nonlinear model parameters, e.g., cubic polynomial, for the whole range of data (*b*_0_, *b*_1_, *b*_2_).
  Define the coordinates of the linear and cubic model intersection point. (*t*_I_ and *t*_II_).
  Estimate linear model parameters (*b*_0_ and b_1_) individually for each of the three segments.
    The advantage of a linear model is the ease of its interpretation. Values of regression indices *b*_1_ present weekly increase of the analysed size *Y*.
3. Polynomial (Fig. 2A): *Y* − *t* characteristics are described with the use of a function in the form of a polynomial:
  $$y(t) = b_{0} + b_{1} \cdot t + b_{2} \cdot t^{2} + \ldots + b_{n} \cdot t_{n}^{n} .$$

**Fig. 2:**
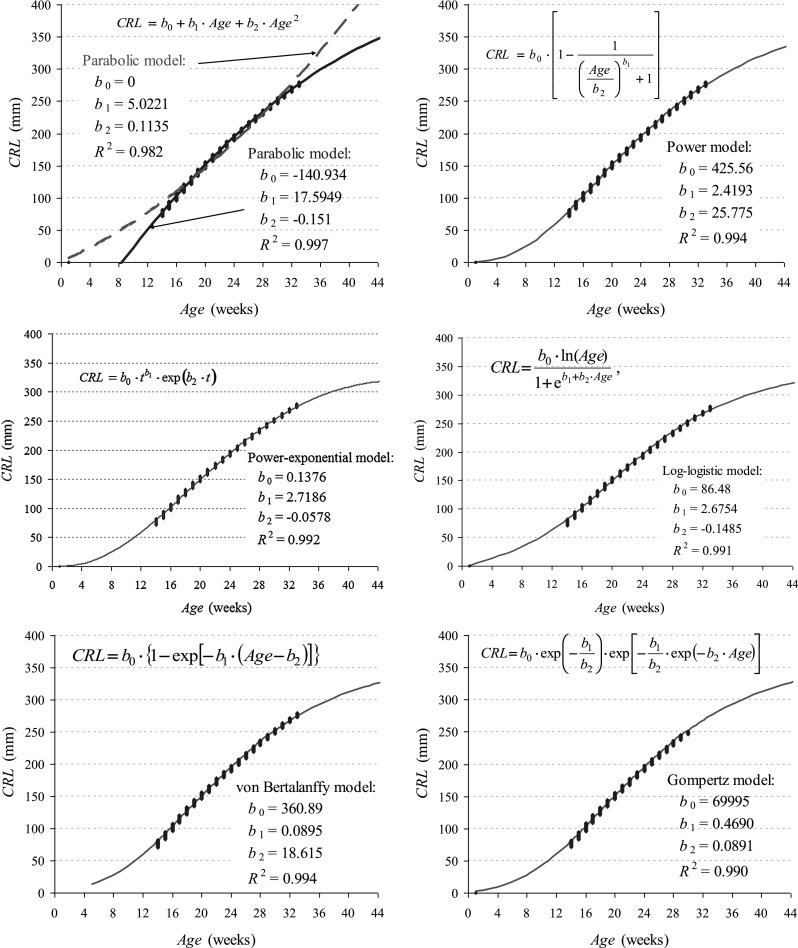
Monoequation, three-parametric models of CRL growth vs. foetal sizes in Scammon’s and Calkins’ tables
4. Spline: the model is composed of segments in which third order curves are matched to the survey results with the smallest squares method.
5. Logarithmic:
  $$y(t) = b_{0} + b_{1} \cdot \ln (t)$$,
  where *b*_1_ > 0.
6. Power (Fig. 2b).
7. Exponential: the function is defined with the following formula:
  $$y(t) = \exp \left( {b_{0} + \frac{{b_{1} }}{t}} \right).$$
8. Power-exponential (Fig. 2c).
9. Logarithmic-logistic (Fig. 2d).
10. Gompertz’s (Fig. 2f).
11. von Bertalanffy’s (Fig. 2e).

The above functions can be further modified by adding successive elements. None of the above growth functions is a universal growth principle. The shape of the growth curve presents a sort of sublimated course characteristic for an average individual of the examined population.

During mathematical model construction, model graphic representation proved to be very helpful. Hence, as the first step, a correlation diagram should be constructed. On the basis of *y*(*t*) − *t* results dispersion, the equation form is adopted and (*b*_0_ *, b*_1_ *…*) parameters values are selected. Such a graph allows one to eliminate ambiguous results that are distant from “the cloud of dots” representing foetuses.

Due to a modelled object (human foetus), the following factors should be considered while mathematical function matching. The model should well describe the sizes of the analysed anatomical structures in the whole observed period, from the first until the 42nd week of foetal life (and even up to the 48th week). All sizes at *t* = 1 should be close to zero (Fig. 1). Such a model enables comparison of many surveys focusing on foetal development during various time intervals of pregnancy.

The applied method of anatomical structure measurement (foetus ultrasound measurement, section material direct measurement), type of tissue (bone, muscle) as well as the size largely influence measurement errors.

A linear model is the simplest but also the least accurate. However, in the case of poor correlation of *y* measurement with age *t* (*r* < 0.5), this choice is well grounded. However, it is necessary to be precise about which period of foetal life its construction refers to. Extrapolation out of the examined period is very risky.

In the case of stronger correlations (*R* ^2^ > 0.85) when the dispersion graph points at growth of a nonlinear character, monotonically increasing functions should be considered. Polynomials application enables model extrapolation out of the examined period of foetal life (in extreme cases, linear sizes may decrease).

Own examinations (Dudek et al. 2014; Kędzia et al. 2010, 2011a, b, 2012, 2013; Woźniak et al. 2012, 2014) show that revealed dependencies *y*(*t*) − *t* can be described accurately enough with one mathematical function only. Most often, exponential, power, logarithmic-logistic, Gompertz’s and von Bertalanffy’s models proved to have the best matching measurement results.

Mathematical analysis of human anatomical structure growth should finally arrive at model (b) parameters estimation. These parameter estimations are achieved by objective function minimization (Jaworski et al. 1992; Lee et al. 2014)

$$S = \sum\limits_{i = 1}^{i = n} {\left( {y_{\,i,meas} - y_{\,i,cal} } \right)\,^{2} } .$$

where: *i*—foetus number; *y*_*i,*meas_—measurement result; *y*_*i,*cal_—calculation result based on mathematical model. Calculations may be carried out with the use of the smallest squares method or Marquardt’s method. The authors use STATISTICA v.10 computer package (StatSoft, Inc. Tulsa, USA).

Determination index *R* ^2^ is usually adopted as a criterion of goodness of fit of a model to measurement results. The highest value of *R* ^2^ is a determinant factor in making the choice of anatomical structure model. In the case of two or more models of *R* ^2^ with similar value, the final choice should be based on the result of “the remainders” distribution analysis—the difference between measurement and theoretical (model) values. Their distribution should be close to normal and should not correlate with foetal age.

In the case of a large amount of data, the model should take into consideration the modifying influence of the environment, which may inhibit or stimulate foetal growth.

## Materials and methods

Section material consisted of rectus abdominis muscle of 67 foetuses and biceps femoris muscle of 75 foetuses (Table 1). The following methods were incorporated into the study: preparation and anthropologic methods, image digital acquisition, Image J computer system measurements and statistical analysis method. We used an anthropologic method based on age determination with the use of crown-rump length—CRL (V-TUB) by Scammon and Calkins (Scammon and Calkins 1929). Studies were conducted on post mortem material and approved by the ethical committee.

**Table 1:** Statistics characterizing examined foetuses

Variable	Group I (rectus abdominis m.) *N* = 75	Group II (biceps femoris m.) *N* = 67
Age (weeks)		
*M* ± SD	21.5 ± 2.0	22.4 ± 2,1
Me (Q_1_; Q_3_)	22 (21; 23)	22 (21; 24)
Min ÷ Max	17 ÷ 26	18 ÷ 28
*V*-*PL* (mm)		
*M* ± SD	240 ± 36	256 ± 32
Me (Q_1_; Q_3_)	245 (220; 263)	252 (233; 278)
Min ÷ Max	132 ÷ 310	191 ÷ 334
CRL (mm)		
*M* ± SD	166 ± 22	177 ± 22
Me (Q_1_; Q_3_)	170 (158; 180)	175 (161; 189)
Min ÷ Max	110 ÷ 212	130 ÷ 237
Body mass (g)		
*M* ± SD	313 ± 117	316 ± 112
Me (Q_1_; Q_3_)	310 (245; 375)	312 (247; 379)
Min ÷ Max	85 ÷ 619	98 ÷ 622
*n* (%) female foetuses	22 (29.3 %)	33 (49.3 %)

*M* mean, *SD* standard deviation, *Me* median, *Q*_1_ lower quartile, *Q*_3_ upper quartile, *Min* minimum, *Max* maximum, *N* number, (%) percentage

## Results

The choice of mathematical function should be based on a real course of the curve presenting growth of anatomical structure linear size *Ύ* in subsequent weeks *t* of pregnancy. Size changes can be described with a segmental-linear model or one-function model with accuracy adequate enough for clinical purposes. The interdependence of size–age is described with many functions. However, the following functions are most often considered: linear, polynomial, spline, logarithmic, power, exponential, power-exponential, log-logistic I and II, Gompertz’s I and II and von Bertalanffy’s function. With the use of procedures described above, mathematical models parameters were assessed for V-PL (the total length of body) and CRL body lengths increases, rectus abdominis total length *h* and its segments *h*I, *h*II, *h*III, *h*IV as well as biceps femoris length and width of long head (LHL and LHW) and of short head (SHL and SHW).

### Example of foetus CRL length increase model

Graphs (Fig. 1) present parameters of analysed monoequation, biparametric (*b*_0_ and *b*_1_) mathematical models for CRL of the trunk (*verte*-*tuberale*). Approximation was made on the basis of CRLs included in Scammon’s and Calkins’ tables (Bożiłow and Sawicki 1980; Grzonkowska et al. 2014). Extrapolation of a linear model from the first weeks of life results in negative values (Fig. 1—green line) and assumption of value zero for the stable expression *b*_0_ reduces the value of *R* ^2^—determination value to nonacceptable values (red line). In turn, an exponential model meets the requirements described above.

Among the examined models with three parameters (*b*_0_, *b*_1_ i *b*_2_), power (*R* ^2^ = 0.994), von Bertallanfy’s (*R* ^2^ = 0.994) and power-exponential (*R* ^2^ = 0.992) models reveal the best adjustment to Scammon’s and Calkins’ tables—Fig. 2.

**Fig. 3:**
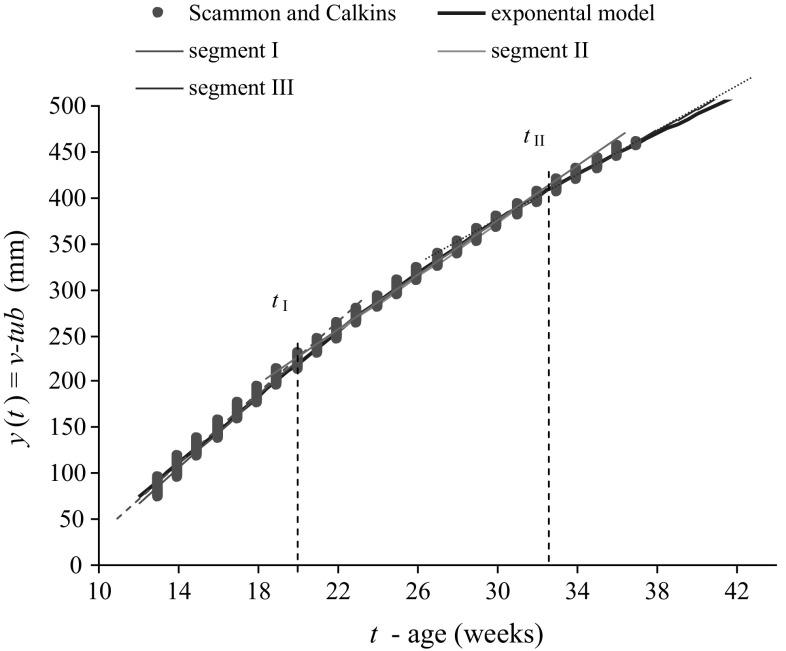
Models of *v*-*tub* length growth: three-equation linear models vs. foetal sizes in Scammon’s and Calkins’ tables

Three-equation linear models are simple to interpret but difficult to construct (Fig. 3). They can be used in measurement data involving a long foetal period (at least 10–40 weeks) (Table 1).

### Mathematical models of rectus abdominis and biceps femoris muscle sizes increase

The above rules of mathematical model construction have been applied to describe the increase in size of rectus abdominis and biceps femoris muscles. Section material consisted of 67 foetuses (rectus abdominalis muscle) and 75 foetuses (biceps femoris muscle).

**Fig. 4:**
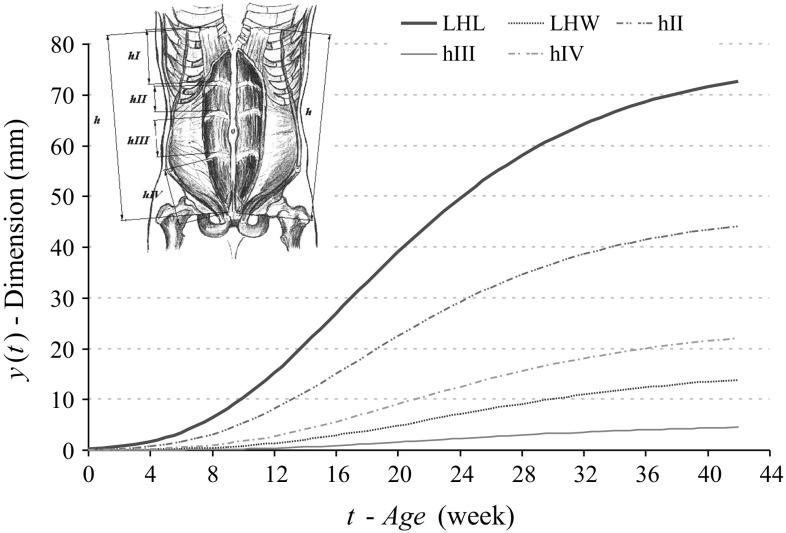
Gompertz’s curves (model 12) illustrating development of analysed parameters of rectus abdominis muscle sizes

**Fig. 5:**
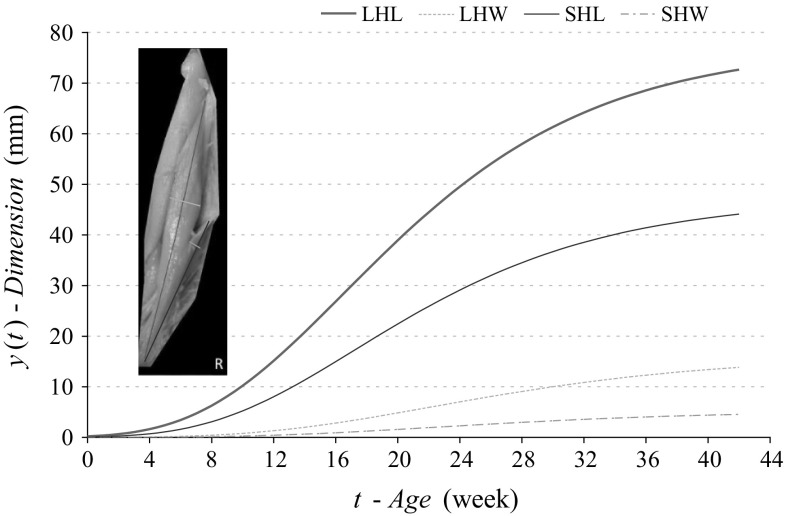
Gompertz’s curves (model 12), illustrating development of analysed sizes of femoral musculus adductor longus

**Fig. 6:**
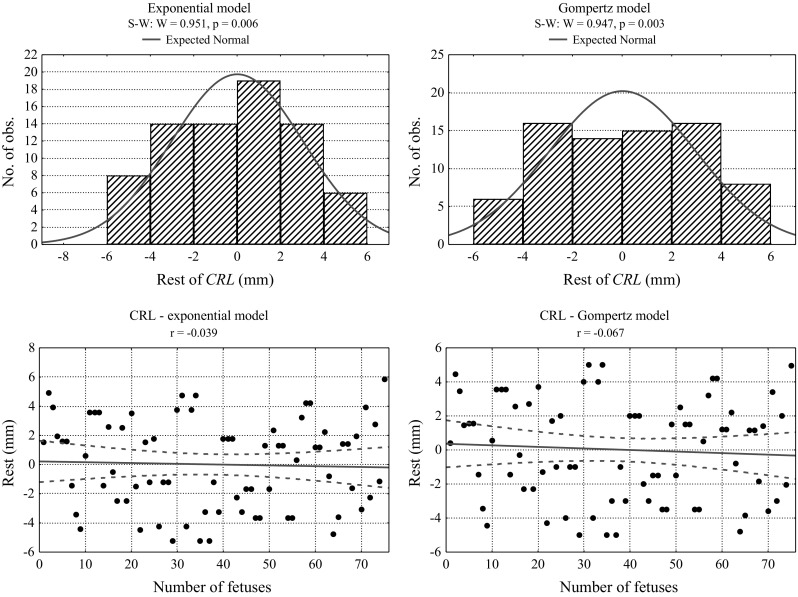
Histograms and correlation diagrams of the rest of the growth models for CRL lengths: exponential and Gompertz’s models

To estimate parameters of mathematical models for length increases of V-PL and CRL, rectus abdominis muscle total length—*h* as well as its segments *h*I, *h*II, *h*III, *h*IV (*G*-*OP*) (Table 2), functions discussed earlier were applied. Gompertz’z model proved the best match with the measurement results (Fig. 4). Table 3 presents parameters of compared mathematical models of biceps femoris muscle-long and short head lengths (LHL and SHL) as well as its widths (LHW and SHW) (Figs. 5, 6).

**Table 2:** Growth model parameters for selected dimensions of rectus abdominis muscle (75 foetuses)

Dimensions (mm)	Model					
	(1) Linear	(6) Power	(7) exponential	(9) log-logistic	(10) Gompertz	(11) von Bertalanffy
V-PL	*b*_0_ = 0 *b*_1_ = 11.189 *R* ^2^ = 0.846	*b*_0_ = 323.452 *b*_1_ = 4.8361 *b*_2_ = 17.106 *R* ^2^ = 0.860	*b*_0_ = 6.8323 *b*_1_ = −29.010 *R* ^2^ = 0.832	*b*_0_ = 126.95 *b*_1_ = 2.2624 *b*_2_ = −0.1272 *R* ^2^ = 0.853	*b*_0_ = 30,210 *b*_1_ = 0.2417 *b*_2_ = 0.0629 ***R*** ^**2**^ **= 0.867**	*b*_0_ = 560.27 *b*_1_ = 0.0751 *b*_2_ = 19.305 *R* ^2^ = 0.860
CRL	*b*_0_ = 0 *b*_1_ = 7.753 *R* ^2^ = 0.911	*b*_0_ = 227.847 *b*_1_ = 4.7726 *b*_2_ = 17.282 *R* ^2^ = 0.919	*b*_0_ = 6.4812 *b*_1_ = −29.352 *R* ^2^ = 0.964	*b*_0_ = 82.40 *b*_1_ = 2.4566 *b*_2_ = −0.1448 *R* ^2^ = 0.964	*b*_0_ = 30,242 *b*_1_ = 0.3381 *b*_2_ = 0.0773 ***R*** ^**2**^ = **0.972**	*b*_0_ = 348.92 *b*_1_ = 0.0868 *b*_2_ = 18.031 *R* ^2^ = 0.968
*H*	*b*_0_ = 0 *b*_1_ = 2.109 *R* ^2^ = 0.466	*b*_0_ = 53.317 *b*_1_ = 6.0390 *b*_2_ = 15.797 *R* ^2^ = 0.466	*b*_0_ = 4.8450 *b*_1_ = −22.070 ***R*** ^**2**^ = **0.472**	*b*_0_ = 17.37 *b*_1_ = 2.9855 *b*_2_ = −0.2227 *R* ^2^ = 0.466	*b*_0_ = 30,226 *b*_1_ = 0.8134 *b*_2_ = 0.1325 *R* ^2^ = 0.472	*b*_0_ = 1105.4 *b*_1_ = 0.0152 *b*_2_ = 98.118 *R* ^2^ = 0.450
*Hi*	*b*_0_ = 0 *b*_1_ = 0.452 *R* ^2^ = 0.190	*b*_0_ = 11.418 *b*_1_ = 5.8449 *b*_2_ = 16.532 *R* ^2^ = 0.204	*b*_0_ = 3.7293 *b*_1_ = −32.157 ***R*** ^**2**^ = **0.221**	*b*_0_ = 4.12 *b*_1_ = 2.7002 *b*_2_ = −0.1976 *R* ^2^ = 0.175	*b*_0_ = 30,230 *b*_1_ = 0.8723 *b*_2_ = 0.1167 *R* ^2^ = 0.220	*b*_0_ = 930.98 *b*_1_ = 0.0150 *b*_2_ = 123.716 *R* ^2^ = 0.198
*h*II	*b*_0_ = 0 *b*_1_ = 0.445 *R* ^2^ = 0.300	*b*_0_ = 10.883 *b*_1_ = 6.7511 *b*_2_ = 15.698 *R* ^2^ = 0.269	*b*_0_ = 3.2959 *b*_1_ = −22.222 *R* ^2^ = 0.301	*b*_0_ = 3.71 *b*_1_ = 2.7923 *b*_2_ = −0.2096 *R* ^2^ = 0.303	*b*_0_ = 30,204 *b*_1_ = 1.1494 *b*_2_ = 0.1487 ***R*** ^**2**^ = **0.310**	*b*_0_ = 111.05 *b*_1_ = 0.020 *b*_2_ = 66.370 *R* ^2^ = 0.293
*h*III	*b*_0_ = 0 *b*_1_ = 0.477 *R* ^2^ = 0.223	*b*_0_ = 17.511 *b*_1_ = 3.4844 *b*_2_ = 19.371 *R* ^2^ = 0.248	*b*_0_ = 3.6173 *b*_1_ = −27.721 *R* ^2^ = 0.240	*b*_0_ = 9.66 *b*_1_ = 2.1665 *b*_2_ = −0.0706 *R* ^2^ = 0.239	*b*_0_ = 30,200 *b*_1_ = 0.9642 *b*_2_ = 0.1284 ***R*** ^**2**^ = **0.253**	*b*_0_ = 116.5 *b*_1_ = 0.0251 *b*_2_ = 57.015 ***R*** ^**2**^ = **0.253**
*h*IV	*b*_0_ = 0 *b*_1_ = 0.615 *R* ^2^ = 0.176	*b*_0_ = 13.860 *b*_1_ = 6.8177 *b*_2_ = 14.565 *R* ^2^ = 0.256	*b*_0_ = 3.9726 *b*_1_ = −35.256 *R* ^2^ = 0.244	*b*_0_ = 4.56 *b*_1_ = 2.7110 *b*_2_ = −0.2526 *R* ^2^ = 0.253	*b*_0_ = 30,210 *b*_1_ = 0.6892 *b*_2_ = 0.0970 ***R*** ^**2**^ = **0.258**	*b*_0_ = 14.46 *b*_1_ = 0.1178 *b*_2_ = 12.003 *R* ^2^ = 0.197

Bold values indicate the best fit model to measured data (the largest value R)

**Table 3:** Growth model parameters for selected dimensions of biceps femoris muscle (67 foetuses)

Dimensions (mm)	Model					
	(1) Linear	(6) Power	(7) exponential	(9) log–logistic	(10) Gompertz	(11) von Bertalanffy
The total length of body: (V-PL)	*b*_0_ = 0 *b*_1_ = 11.444 *R* ^2^ = 0.801	*b*_0_ = 335.281 *b*_1_ = 5.1305 *b*_2_ = 17.705 *R* ^2^ = 0.940	*b*_0_ = 6.8722 *b*_1_ = −29.670 ***R*** ^**2**^ = **0.957**	*b*_0_ = 128.54 *b*_1_ = 2.2354 *b*_2_ = −0.1257 *R* ^2^ = 0.947	*b*_0_ = 30,214 *b*_1_ = 0.2552 *b*_2_ = 0.0657 *R* ^2^ = 0.933	*b*_0_ = 560.28 *b*_1_ = 0.0757 *b*_2_ = 19.158 *R* ^2^ = 0.947
Crown-rump length (CRL)	*b*_0_ = 0 *b*_1_ = 7.914 *R* ^2^ = 0.867	*b*_0_ = 225.302 *b*_1_ = 5.3402 *b*_2_ = 17.468 *R* ^2^ = 0.968	*b*_0_ = 6.5003 *b*_1_ = −29.603 *R* ^2^ = 0.993	*b*_0_ = 98.81 *b*_1_ = 2.0357 *b*_2_ = −0.1045 *R* ^2^ = 0.982	*b*_0_ = 30,809 *b*_1_ = 0.3455 *b*_2_ = 0.0785 ***R*** ^**2**^ = **0.997**	*b*_0_ = 462.23 *b*_1_ = 0.0609 *b*_2_ = 21.743 *R* ^2^ = 0.982
Long head length (LHL)	*b*_0_ = 0 *b*_1_ = 2.027 *R* ^2^ = 0.692	*b*_0_ = 62.925 *b*_1_ = 4.8755 *b*_2_ = 18.231 *R* ^2^ = 0.683	*b*_0_ = 5.9690 *b*_1_ = −42.529 *R* ^2^ = 0.802	*b*_0_ = 18.53 *b*_1_ = 3.4922 *b*_2_ = −0.2164 *R* ^2^ = 0.716	*b*_0_ = 30,199 *b*_1_ = 0.3537 *b*_2_ = 0.0789 ***R*** ^**2**^ = **0.834**	*b*_0_ = 68.71 *b*_1_ = 0.1439 *b*_2_ = 16.131 *R* ^2^ = 0.575
Long head width (LHW)	*b*_0_ = 0 *b*_1_ = 0.242 *R* ^2^ = 0.692	*b*_0_ = 10.648 *b*_1_ = 4.1047 *b*_2_ = 21.849 *R* ^2^ = 0.835	*b*_0_ = 3.0947 *b*_1_ = −31.409 *R* ^2^ = 0.716	*b*_0_ = 2.06 *b*_1_ = 6.8092 *b*_2_ = −0.3881 *R* ^2^ = 0.462	*b*_0_ = 30,738 *b*_1_ = 0.6887 *b*_2_ = 0.0913 *R* ^2^ = 0.848	*b*_0_ = 7.09 *b*_1_ = 0.2967 *b*_2_ = 17.151 *R* ^2^ = 0.213
Short head length (SHL)	*b*_0_ = 0 *b*_1_ = 1.186 *R* ^2^ = 0.271	*b*_0_ = 38.508 *b*_1_ = 3.9682 *b*_2_ = 17.575 *R* ^2^ = 0.519	*b*_0_ = 4.6774 *b*_1_ = −31.168 ***R*** = **0.748**	*b*_0_ = 10.66 *b*_1_ = 3.3086 *b*_2_ = −0.2177 *R* ^2^ = 0.537	*b*_0_ = 30,806 *b*_1_ = 0.6998 *b*_2_ = 0.1080 *R* ^2^ = 0.736	*b*_0_ = 33.83 *b*_1_ = 0.2056 *b*_2_ = 14.516 *R* ^2^ = 0.565
Short head width (SHW)	*b*_0_ = 0 *b*_1_ = 0.090 *R* ^2^ = 0.356	*b*_0_ = 2.539 *b*_1_ = 5.5010 *b*_2_ = 17.232 *R* ^2^ = 0.713	*b*_0_ = 1.8310 *b*_1_ = −25.163 *R* = 0.660	*b*_0_ = 0.89 *b*_1_ = 2.0111 *b*_2_ = −0.1341 *R* ^2^ = 0.359	*b*_0_ = 11,403 *b*_1_ = 0.6996 *b*_2_ = 0.0913 ***R*** ^**2**^ = **0.741**	*b*_0_ = 3.62 *b*_1_ = 0.0871 *b*_2_ = 16.141 *R* ^2^ = 0.359

Bold values indicate the best fit model to measured data (the largest value R)

A histogram of the rest of CRL lengths (variance between the model and the measurement), as well as a correlation diagram of the rest of the foetuses arranged in ascending order with reference to age, reveal minimal predominance of the exponential model over Gompertz’s one. The minimally larger convergence of exponential model rests with normal distribution in comparison with Gompertz’s distribution (0.006 vs. 0.003), and the primarily smaller number of parameters of the model (2 vs. 3) level a small difference of *R* ^2^ determination index (0.993 vs. 0.997).

## Discussion

In their surveys, Szpinda et al. (Szpinda et al. 2011) studied musculus biceps femoris and defined its increase in foetuses aged 17–30 weeks with the use of linear function. No significant sex differences were found (*p* > 0.05). All the parameters were found to increase in a linear fashion during gestation and significant positive correlations were found. There were significant laterality differences only in relation to either parameter of the short head of the biceps femoris.

In the studies concerning muscular development, which were carried out on the material of 30 foetuses aged 17–30 weeks of foetal life, linear function was sufficient to describe development dynamics of the following muscles: *triceps brachii* (Grzonkowska et al. 2014), *semimembranosus* (Badura et al. 2011a), *semitendinosus* (Badura et al. 2011a) and *biceps brachii* (Szpinda et al. 2013), due to their comparatively small sizes and large dispersion results.

Neither male–female nor right–left differences are observed in morphometric parameters of the triceps brachii muscle (Grzonkowska et al. 2014). The long head’s belly is the thinnest, while the lateral head’s belly is the widest. The long head is the longest and the medial head is the shortest. The developmental dynamics of the triceps brachii muscle follow proportionately.

**Fig. 7:**
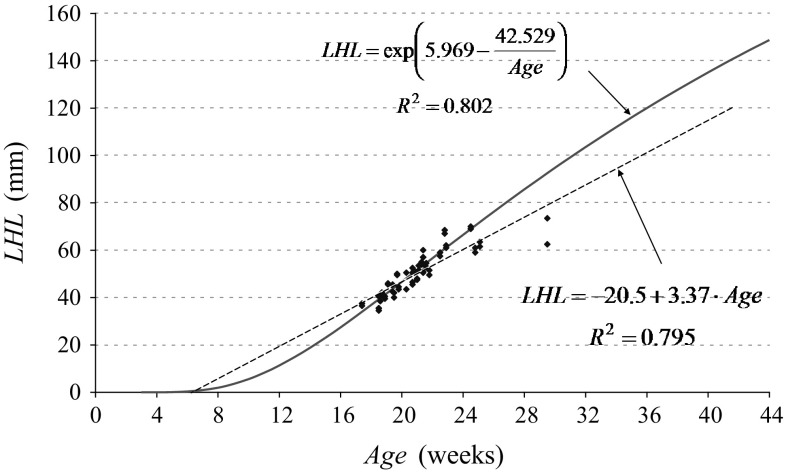
Parameters of linear and exponential models of the growth of musculus biceps femoris long head assessed on the basis of sectional material (Kędzia et al. 2012)

In our own studies, growth dynamics proceeded along with exponential model. Figure 7 reveals that an exponential model is better adjusted to measurement results (0.802 vs. 0.795) and can be extrapolated towards younger foetuses. In the case of a linear model, the lengths have negative values from the first to the sixth week. Linear model can be applied in foetuses only from the 17th to the 29th week, and in the case of exponential model, foetal sizes are always bigger than 0 and they can be applied for the entire foetal period. Adoption of the proposed models will allow other researchers to carry out meta analysis. However, the studies should be broadened from the 29th to the 42nd week (in ultrasound examinations).

Results of nasal cavity geometrical measurements from 138 human foetal head sections aged 14–28 weeks of foetal life were analysed statistically (Kędzia et al. 2013). The measurements were made on 68 left and 70 right halves. Mathematical models were constructed based on nonlinear models. Considered functions were: logarithmic function and Gompertz’s function. Gompertz’s model proved best at matching the measurement results. Nasal cavity anatomical structures increase more quickly between the 14th and 20th weeks and then the growth rate decreases. Neither sexual dimorphism nor anatomical structure asymmetry was observed. Apart from medial nasal turbinate, the growth is steady and gradual in all directions (Kędzia et al. 2013).

The study examined 220 human brachial plexuses, derived from 110 fetuses (including 50 females—45.45 %) aged 14–32 weeks of fetal life, with a crown-rump length (CRL) ranging from 80 to 233 mm (Woźniak et al. 2012). The prenatal development of the brachial plexus was not constant; the applied mathematical functions proved useful in describing its growth rate. Four formulas were used in the mathematical growth model: linear regression, logarithmic function, the von Bertalanffy growth model and the Gompertz curve (Woźniak et al. 2012).

The goal of this study was the mathematical assessment of foetal age with the use of thorax selected dimensions (Woźniak et al. 2014). The material consisted of 110 foetuses aged 4–7 months of foetal life, including 50 females in the CRL range: 80–233 mm. Foetus biometrics allows us to assess the mathematical relation between gestational age foetus biometric parameters. Six monofunctional mathematical models were elaborated: a Bertalanffy growth curve, three Gompertz function based models and two exponential models to assess examined parameters increase along with *t* age (Woźniak et al. 2014).

Gompertz’s model has been used to define life expectancy in elderly people (Ekonomov and Larygin 1989; Lee et al. 2014) as well as in experimental oncology as far as tumour growth was concerned (Hartung et al. 2014). This survey's practical value is based on its applicability in foetus age assessment in ultrasound examination. The proposed models of foetal structure increase, constructed on the basis of new computer techniques and objectively high-tech mathematical calculations, allow us to fill the blanks in the present literature.

## Conclusions

Human foetal anatomical structure changes can be described accurately enough for clinical and prognostic purposes with segmental-linear models or one-function models. The degree of adjustment of model parameters and measurement results is influenced by the function form and especially the structure size absolute value. For bigger structures, e.g., femoral musculus adductor longus, determination index is comprised within the range 58–83 %, whereas in the case of smaller structures, e.g., musculus adductor longus width, the *R* ^2^ value amounts to 52–75 %.
